# *Brucella abortus*-Stimulated Platelets Activate Brain Microvascular Endothelial Cells Increasing Cell Transmigration through the Erk1/2 Pathway

**DOI:** 10.3390/pathogens9090708

**Published:** 2020-08-27

**Authors:** Ana María Rodríguez, Aldana Trotta, Agustina P. Melnyczajko, M. Cruz Miraglia, Kwang Sik Kim, M. Victoria Delpino, Paula Barrionuevo, Guillermo Hernán Giambartolomei

**Affiliations:** 1 Instituto de Inmunología, Genética y Metabolismo (INIGEM), CONICET, Facultad de Farmacia y Bioquímica, Universidad de Buenos Aires, Buenos Aires C1120AAD, Argentina; amrodriguez@fmed.uba.ar (A.M.R.); amelnyczajko@uade.edu.ar (A.P.M.); miraglia.maria@inta.gob.ar (M.C.M.); mdelpino@ffyb.uba.ar (M.V.D.); 2 Instituto de Medicina Experimental (IMEX) (CONICET-Academia Nacional de Medicina), Buenos Aires C1425ASU, Argentina; aldana.trotta@unahur.edu.ar (A.T.); pbarrionuevo@fbmc.fcen.uba.ar (P.B.); 3 Division of Pediatric Infectious Diseases, Department of Pediatrics, Johns Hopkins University School of Medicine, Baltimore, MD 21287, USA; kwangkim@jhmi.edu

**Keywords:** *Brucella abortus*, neurobrucellosis, platelets, brain microvascular endothelial cells, endothelial cells

## Abstract

Central nervous system invasion by bacteria of the genus *Brucella* results in an inflammatory disorder called neurobrucellosis. A common feature associated with this pathology is blood–brain barrier (BBB) activation. However, the underlying mechanisms involved with such BBB activation remain unknown. The aim of this work was to investigate the role of *Brucella abortus*-stimulated platelets on human brain microvascular endothelial cell (HBMEC) activation. Platelets enhanced HBMEC activation in response to *B. abortus* infection. Furthermore, supernatants from *B. abortus*-stimulated platelets also activated brain endothelial cells, inducing increased secretion of IL-6, IL-8, CCL-2 as well as ICAM-1 and CD40 upregulation on HBMEC compared with supernatants from unstimulated platelets. Outer membrane protein 19, a *B. abortus* lipoprotein, recapitulated *B. abortus*-mediated activation of HBMECs by platelets. In addition, supernatants from *B. abortus*-activated platelets promoted transendothelial migration of neutrophils and monocytes. Finally, using a pharmacological inhibitor, we demonstrated that the Erk1/2 pathway is involved in the endothelial activation induced by *B. abortus*-stimulated platelets and also in transendothelial migration of neutrophils. These results describe a mechanism whereby *B. abortus*-stimulated platelets induce endothelial cell activation, promoting neutrophils and monocytes to traverse the BBB probably contributing to the inflammatory pathology of neurobrucellosis.

## 1. Introduction

Blood–brain barrier (BBB) integrity is necessary to protect the brain from injuries such as toxins and germs, as well as to help in maintaining central nervous system (CNS) homeostasis [1]. BBB activation and dysfunction contributes to several brain pathologies. Many factors are able to induce BBB dysfunction such as inflammatory mediators, matrix metalloproteinases, free radicals, and vascular endothelial growth factor, among others [2].

Bacteria of the genus *Brucella* produce several types of inflammatory disorders [3]. Neurobrucellosis is a neurodegenerative inflammatory disorder caused by invasion of the CNS by *Brucella* spp. and constitutes the most morbid pathology associated with this infection [4]. One of the most characteristic clinical signs of this disease is pleocytosis; i.e., the presence of leukocytes in the cerebrospinal fluid [4].

We have recently described a putative mechanism employed by *Brucella abortus* to gain access to the CNS. We have demonstrated, using an in vitro model, that *B. abortus* traverses the BBB into the cerebral parenchyma inside infected monocytes, by a mechanism known as “Trojan horse”. Moreover, infected monocytes act as bacterial source for de novo infection of glial cells [5]. In addition, we have described that activation of glial cells by *B. abortus* is crucial in neurobrucellosis pathology [6]. *B. abortus*-activated astrocytes and microglia secrete pro-inflammatory mediators such as tumor necrosis factor (TNF)-α, interleukin (IL)-6, IL-1β, C-C motif chemokine ligand 2 CCL-2, C-X-C motif chemokine ligand 1 (CXCL1), metalloproteinase (MMP)-9, and nitric oxide (NO), among others [6,7,8]. These inflammatory mediators, and astrocytes/microglia-secreted IL-1β in particular, activate the BBB, allowing monocyte and neutrophil transmigration [9]. The effect of glial cell activation on BBB cells is well known [9,10,11]. However, whether peripheral inflammation induced by *Brucella*-activated cells can modify the BBB remains unknown.

Platelets are well characterized as responsible for maintaining vascular integrity in addition to being hemostatic mediators [12]. In the last few years, the immune function of platelets has been described in both homeostasis and pathology [12,13,14]. Platelets express several immune receptors such as toll-like receptors (TLR) and Fc receptors, which allow the recognition of different pathogens [12]. Upon pathogen recognition, platelets can be activated and secrete a wide variety of immunomodulatory mediators present in their granules [15,16,17]. The immunoregulatory functions of pathogen-activated platelets have been described recently, as well as their ability to activate endothelial cells, including the microvascular endothelium of the BBB [14,18,19,20]. We have recently described the interactions between platelets and *B. abortus* [14]. *B. abortus* is able to invade, infect platelets, and activate them. As a consequence of this interaction, platelets establish complexes with *B. abortus*-infected monocytes, increasing the efficiency of the infection and modulating monocyte and neutrophil functions [14,21]. Moreover, we demonstrated that complexes between platelets and both monocytes and neutrophils are more abundant in patients with brucellosis than in healthy donors [21].

We have already described the effect of glial cell activation on BBB cells during neurobrucellosis; however, whether platelets activated by *Brucella* can modify the BBB remains unexplored. Therefore, the aim of this work was to elucidate whether *B. abortus*-activated platelets can activate the BBB and affect transmigration of monocytes and neutrophils. Here, we demonstrated that *B. abortus*-activated platelets activate brain microvascular endothelial cells, and other endothelial cells, through Erk1/2 signaling pathway, leading monocytes and neutrophils to traverse polarized brain microvascular endothelial monolayers.

## 2. Results

### 2.1. Interaction with Platelets Enhances the Activation of Endothelial Cells in the Context of B. abortus Infection

**Figure 1:**
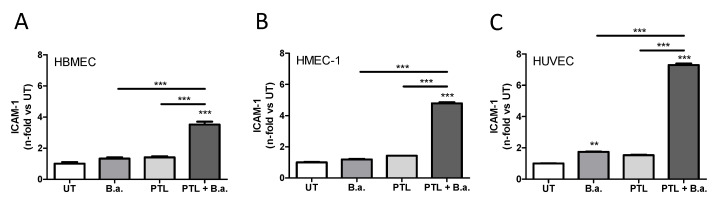
Interaction with platelets enhances the activation of endothelial cells in the context of *Brucella abortus* infection. Endothelial cells lines human brain microvascular endothelial cell (HBMEC) (**A**), human microvascular endothelial cells (HMEC-1) (**B**), and human umbilical vein endothelial cell (HUVEC) primary culture (**C**) were incubated with *B. abortus* (B.a.), in the presence or the absence of platelets (PTL), or with PTL alone for 24 h. ICAM-1 (intercellular adhesion molecule 1) expression on the cell surface was assessed by flow cytometry. Bars represent the mean ± SEM of duplicates. Data shown are from a representative experiment out of at least three performed. ** *p* < 0.005, *** *p* < 0.0005 vs. untreated cells (UT).

We decided to evaluate the capacity of *B. abortus* to activate human brain microvascular endothelial cells (HBMECs) in presence of platelets. For this, HBMECs were co-cultured with platelets (cell:platelet ratio, 1:100) and infected with *B. abortus* (MOI of 100) for 24 h. As control, HBMECs were cultured with platelets alone or they were infected in the absence of platelets. We measured ICAM-1 (intercellular adhesion molecule 1, also known as CD54) expression to determine the level of activation of endothelial cells. Endothelial ICAM-1 plays a critical role at different steps of neutrophil migration into inflamed tissues [22]. As we have previously reported, infection with *B. abortus* in the absence of platelets induced a slight activation of HBMECs, measured as the upregulation of surface ICAM-1 [9]. The presence of platelets in the absence of infection also induced a slight activation of HBMECs. However, these effects were not significant. On the other hand, the presence of platelets during the infection of HBMECs induced a significant (*p* < 0.0005) upregulation of ICAM-1 (Figure 1A). These results demonstrate that the presence of platelets enhances *B. abortus*-induced activation of microvascular brain endothelial cells.

To investigate whether the effect of platelets during *B. abortus* infection also occurs in other endothelia, human microvascular endothelial cells (HMEC-1) and human umbilical vein endothelial cell (HUVEC) were infected with *B. abortus* in the presence or absence of platelets. The presence of platelets during the infection of both types of endothelial cells induced a significant upregulation of ICAM-1 expression compared to infected cells or cells incubated with platelets alone (*p* < 0.0005) (Figure 1B,C). These data demonstrate that the presence of platelets enhances *B. abortus*-activation of different endothelial cell types.

### 2.2. Supernatants from B. abortus-Stimulated Platelets Activate Brain Microvascular Endothelial Cells

**Figure 2:**
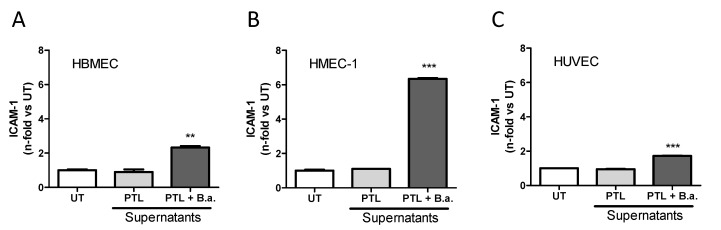
Supernatants from *B. abortus*-stimulated platelets activate endothelial cells. Supernatants collected from *B. abortus*-stimulated platelets (PTL + B.a.) or platelets alone (PTL) were used to stimulate the endothelial cell lines HBMEC (**A**), HMEC-1 (**B**), and HUVEC primary culture (**C**) for 24 h (dilution 1:2). ICAM-1 expression was measured on the cell surface by flow cytometry. ** *p* < 0.005, *** *p* < 0.0001 vs. untreated cells (UT).

To investigate whether this effect was due to direct interaction between platelets and endothelial cells or factors released by *B. abortus*-activated platelets, we performed experiments using conditioned media. First, platelets were stimulated with or without *B. abortus* (platelets: *B. abortus* ratio, 1:1) for 24 h. Then, culture supernatants were collected and filtered to eliminate platelets and bacteria. Finally, cell-free supernatants were used to stimulate HBMECs for an additional 24 h. Stimulation of HBMECs with supernatants from *B. abortus*-stimulated platelets induced a significant (*p* < 0.005) upregulation of ICAM-1 surface expression (Figure 2A). These results demonstrate that supernatants from *B. abortus*-stimulated platelets are able to activate microvascular brain endothelial cells. Furthermore, in order to expand our results, we investigated whether these supernatants could also activate other endothelial cell types. For this, HMEC-1 and HUVEC were stimulated with supernatants collected from *B. abortus*-stimulated platelets. We observed an upregulation of ICAM-1 surface expression on both cell types (Figure 2B,C). Collectively, these data demonstrated that supernatants from *B. abortus*-stimulated platelet are able to activate several types of endothelial cells.

**Figure 3:**
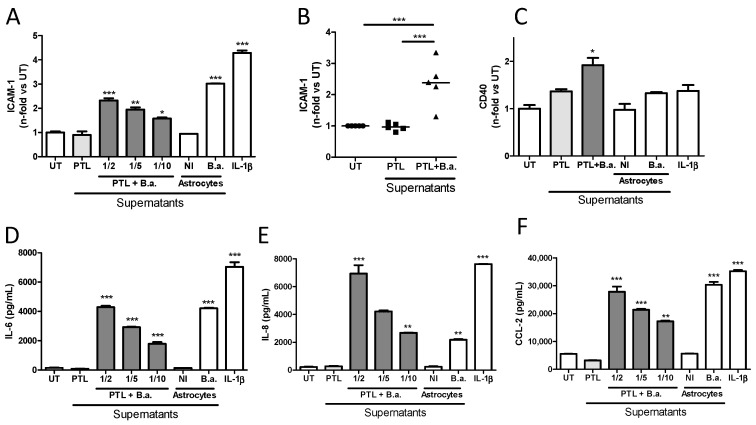
Supernatants from *B. abortus*-infected platelets induce an activated phenotype in HBMECs. Platelets were incubated with *B. abortus* (PTL + B.a.) or alone (PTL) for 24 h. Culture supernatants from *B. abortus*-infected astrocytes and interleukin (IL)-1β were used as control. Platelets supernatants were collected, filtered, and used to stimulate HBMECs for 24 h at the indicated dilutions. (**A**) ICAM-1 expression was measured by flow cytometry on HBMEC surface. (**B**) ICAM-1 expression obtained by stimulating HBMECs with supernatants obtained from five independent PTL donors. (**C**) CD40 expression was measured on HBMEC surface by flow cytometry. The secretion of IL-6 (**D**), IL-8 (**E**), and C-C motif chemokine ligand 2 (CCL-2) (**F**) was determined from HBMEC treated culture supernatants by ELISA. Bars represent the mean ± SEM of duplicates. Data shown are from a representative experiment out of at least three performed, except B. * *p* < 0.05, ** *p* < 0.005, *** *p* < 0.0005 vs. untreated cells (UT). NI: non infected.

Next, we studied in depth the HBMEC activation induced by supernatants from *B. abortus*-stimulated platelets. Activation of HBMECs induced by supernatants from *B. abortus*-stimulated platelets was dose dependent (Figure 3A) and, more importantly, it was achieved by using supernatants from different platelet donors (Figure 3B). CD40 expression in endothelial cells has been implicated in several pathologic conditions of the CNS including Alzheimer’s disease and human immunodeficiency virus 1 (HIV-1) encephalitis, where an important role of CD40 has been demonstrated in BBB disruption [23]. Besides the upregulation of ICAM-1, the activated phenotype induced by *B. abortus*-infected platelet supernatants also included the significant upregulation of CD40 surface expression (*p* < 0.05) (Figure 3C) and the secretion of significant (*p* < 0.0005) amounts of IL-6, IL-8, and CCL-2 (Figure 3D–F) when compared with untreated HBMECs or HBMECs treated with supernatants from unstimulated platelets. Levels of activation were comparable to those obtained when HBMECs were stimulated with culture supernatants from *Brucella*-infected astrocytes [9] or IL-1β used as positive controls. Importantly, the concentrations of IL-6, IL-8, and CCL-2 measured in supernatants from *B. abortus*-stimulated platelets used for stimulation were negligible (<200 pg/mL in all cases, data not shown). These results demonstrate that supernatants from *B. abortus*-stimulated platelets induce an activated phenotype in microvascular brain endothelial cells, characterized by the upregulation of surface molecules such as ICAM-1 and CD40, and the secretion of both cytokines and chemokines.

### 2.3. Secreted Factors from B. abortus-Activated Platelets Activate HBMECs

**Figure 4:**
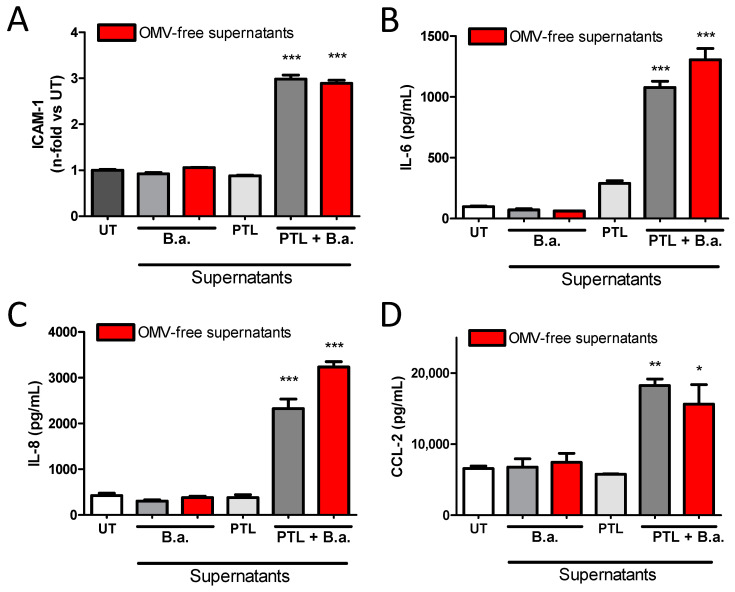
Secreted factors from *B. abortus*-stimulated platelets activate HBMECs. Supernatants from *B. abortus*-activated platelets (PLT + B.a.) or from *B. abortus* alone were ultracentrifuged (outer-membrane vesicle (OMV)-free supernatants) or not and used to stimulate HBMECs for 24 h. Supernatants from non-ultracentrifuged PTL alone were used as control. (**A**) ICAM-1 surface expression on HBMECs was assessed by flow cytometry. HBMEC secretion of IL-6 (**B**), IL-8 (**C**), and CCL-2 (**D**) was quantified by ELISA. Bars represent the mean ± SEM of duplicates from a representative experiment out of at least three performed. * *p* < 0.05, ** *p* < 0.005, *** *p* < 0.0001 vs. untreated cells (UT).

Previous studies have shown that *Brucella* spp. release outer-membrane vesicles (OMVs, also known as blebs) containing lipopolysaccharide (LPS), outer membrane proteins, and other bacterial components [24]. To rule out the possibility that OMVs were implicated in HBMEC activation, they were removed from the supernatants by ultracentrifugation, as previously described [24]. HBMECs were then incubated with OMVs-free supernatants for 24 h and the activation of HBMECs was evaluated. There were no significant differences (*p* > 0.05) between non-depleted and OMVs-free supernatants regarding HBMEC activation, measured as ICAM-1 expression (Figure 4A) and IL-6, IL-8, and CCL-2 secretion (Figure 4B–D, respectively). To discard any putative participation of *Brucella*-secreted factors on HBMEC activation, *B. abortus* was incubated alone in the same culture conditions for 24 h. Then, culture supernatants were filtered and ultracentrifuged as described above. These platelet-free *B. abortus* culture supernatants were unable to activate HBMECs (Figure 4). Altogether, these results indicate that secreted factors from *B. abortus*-activated platelets are responsible for HBMEC activation.

### 2.4. B. abortus Lipoprotein-Stimulated Platelets Activate Brain Microvascular Endothelial Cells

**Figure 5:**
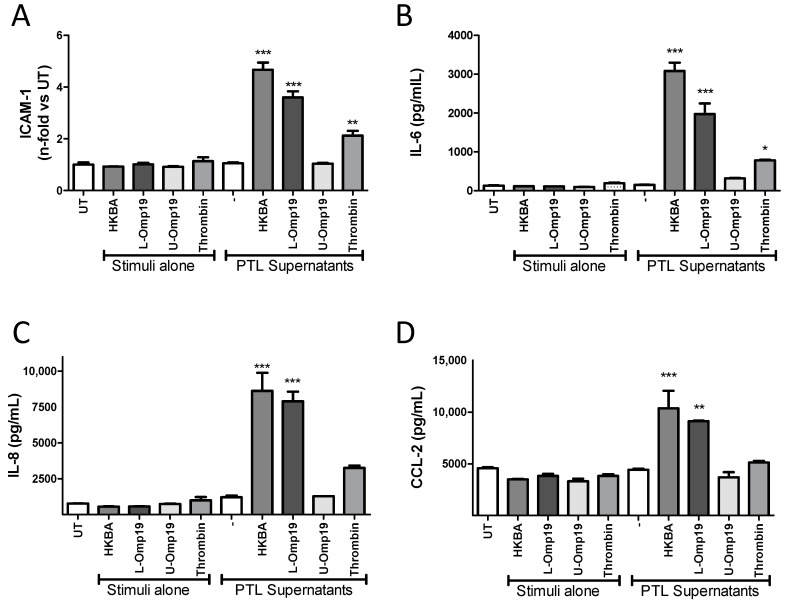
*B. abortus* lipoprotein-stimulated platelets activate HBMECs. Platelets were stimulated for 24 h with heat killed *B. abortus* (HKBA, 10^8^ bacteria/mL), lipidated-outer-membrane protein 19 (L-Omp19), unlipidated-outer-membrane protein 19 (U-Omp19) (500 ng/mL), or thrombin (0.1 U/mL). Then, supernatants were collected, filtered. and used to stimulate HBMECs for an additional 24 h. Supernatants from PTL and stimuli incubated alone at the same conditions were used as control. ICAM-1 (**A**) was determined on HBMEC surface by flow cytometry. The secretion of IL-6 (**B**), IL-8 (**C**), and CCL-2 (**D**) was determined by ELISA. Bars represent the mean ± SEM of duplicates from a representative experiment out of at least three performed. * *p* < 0.05, ** *p* < 0.005, *** *p* < 0.0001 vs. untreated cells (UT).

To test whether bacterial viability was necessary to induce the activation of platelets and consequently HBMEC activation, platelets were incubated for 24 h with heat-killed *B. abortus* (HKBA). Supernatants were then filtered and used as stimuli on HBMECs. HKBA supernatants were used to treat HBMECs as a negative control. As positive control, platelets were activated with thrombin. Supernatants from HKBA-stimulated platelets were able to activate HBMECs, inducing the upregulation of ICAM-1 (Figure 5A), and increasing the secretion of IL-6, IL-8, and CCL-2 (Figure 5B–D, respectively). We have previously demonstrated that different cell types can be activated by *B. abortus* lipoproteins [8,25,26]. Therefore, we further evaluated the contribution of lipoproteins in the induction of HBMEC activation by platelets. For this, platelets were incubated with *B. abortus* lipidated- or unlipidated-outer-membrane protein 19 (L-Omp19 or U-Omp19, respectively), used as a *Brucella* lipoprotein model [25]. Then, HBMECs were stimulated with the filtered supernatants for an additional 24 h, and the expression of ICAM-1 and secretion levels of IL-6, IL-8, and CCL-2 were evaluated. L-Omp19-activated platelets recapitulated HBMEC activation induced by supernatants from *B. abortus*-stimulated platelets. Furthermore, this activation was dependent on the lipidation of Omp19, as U-Omp19-stimulated platelets failed to induce HBMEC activation (Figure 5A–D). Culture supernatants from thrombin-activated platelets also induced partial activation of HBMECs. Neither HKBA-, L-Omp19-, nor U-Omp19-stimulated supernatants were able to activate HBMECs, demonstrating the presence of a platelet-secreted factor involved in the activation of HBMECs. Altogether, these results demonstrated that the presence of supernatants from platelets stimulated by structural components of *Brucella* (such as L-Omp19), independently of bacterial viability, are involved in the activation of HBMECs.

### 2.5. Platelet-Stimulated HBMECs Induce Transendothelial Migration of Neutrophils and Monocytes

The presence of leukocytes in the cerebrospinal fluid and cerebral parenchyma has been described during neurobrucellosis [4]. This phenomenon, named pleocytosis, could be a consequence of the activation induced by *Brucella*-activated platelets on the blood–brain barrier [9]. To test this possibility, we used a previously established assay of transendothelial migration [9]. Briefly, HBMECs were seeded in the upper chamber of a Transwell plate, and they were cultured for 5 days to establish a monolayer. Then, HBMEC monolayers were treated for 24 h with supernatants from *B. abortus*-stimulated platelets. Culture supernatants from *Brucella*-infected astrocytes and human IL-1β were used as control. Finally, neutrophils or monocytes were seeded in the upper chamber and incubated for 3 h and the number of transmigrated cells to the lower chamber was quantified.

**Figure 6:**
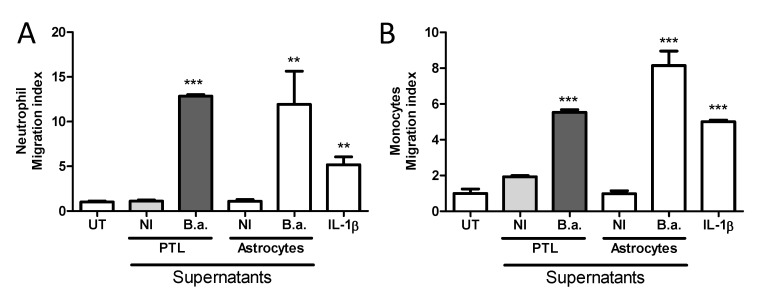
*B. abortus*-infected platelets activate HBMEC, promoting transendothelial migration of neutrophils and monocytes. HBMEC monolayers were established on the membrane of Transwell plates and then treated with supernatants from *B. abortus*-stimulated platelets for additional 24 h. Culture supernatants from *Brucella*-infected astrocytes and human IL-1β were used as control. Next, neutrophils (**A**) or monocytes (**B**) were seeded in the upper chamber and incubated for 3 h. Finally, media from the lower chamber were harvested and the number of migrated cells was quantified. Bars represent the mean ± SEM of duplicates from a representative experiment of at least three performed. ** *p* < 0.005, *** *p* < 0.0005 vs. untreated cells (UT) or indicated treatment. NI: non infected.

Monocyte as well as neutrophil migration increased when the HBMEC monolayer was treated with supernatants from *B. abortus*-stimulated platelets (Figure 6A,B), but not when HBMECs were stimulated with supernatants from platelets alone. Cellular transmigration was comparable to that obtained when HBMECs where stimulated with culture supernatants from *Brucella*-infected astrocytes or IL-1β used as positive controls. These results indicate that activation of brain endothelial cells by supernatants from *B. abortus*-stimulated platelets could induce transmigration of immune cells through a polarized brain endothelial cell monolayer. Taken together, these results suggest that activated platelets could be responsible for the induction of pleocytosis in the context of *B. abortus* CNS infection.

### 2.6. The Erk1/2 Pathway Is Involved in HMBEC Activation Induced by B. abortus-Stimulated Platelets and It Is Implicated in Transendothelial Migration of Neutrophils

**Figure 7:**
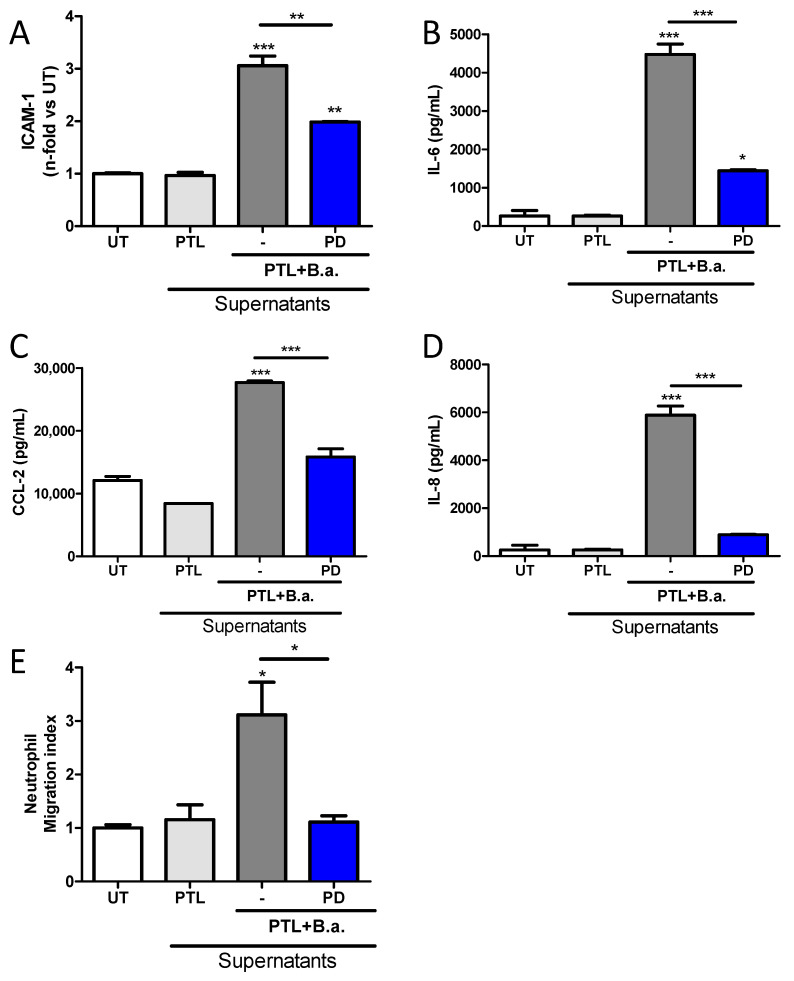
Extracellular signal-regulated kinase (Erk)1/2 pathway is involved in HBMEC activation induced by *B. abortus*-stimulated platelets and it is implicated in transendothelial migration of neutrophils. HBMEC were pre-incubated with the Erk1/2 inhibitor (PD98059) for 2 h before platelets–supernatants stimulation and kept throughout. ICAM-1 was determined on HBMECs surface by flow cytometry (**A**). The secretion of IL-6 (**B**), IL-8 (**C**), and CCL-2 (**D**) was determined by ELISA. HBMEC monolayers were established on the membrane of Transwell plates. HBMEC activation was inhibited by PD98059 and then treated with supernatants from *B. abortus*-stimulated platelets for additional 24 h. Next, neutrophils were seeded in the upper chamber and incubated for 3 h. Finally, media from the lower chamber was harvested and the number of migrated cells was quantified (**E**). Bars represent the mean ± SEM of duplicates from a representative experiment of at least three performed * *p* < 0.05, ** *p* < 0.005, *** *p* < 0.0005 vs. untreated cells (UT) or indicated treatment.

We decided to further investigate the molecular mechanisms involved in endothelial activation and cellular transcytosis. It was previously reported that the extracellular signal-regulated kinase (Erk)1/2 pathway is involved in the activation of brain endothelial cells [27]. Taking this into account, we investigated the participation of the Erk1/2 signaling pathway in the activation of HBMECs by supernatants from *B. abortus*-stimulated platelets. For this, we used the Erk1/2-specific inhibitor PD98059 to treat HBMEC cells. Inhibition of the Erk1/2 pathway partially reduced the upregulation of ICAM-1 induced by supernatants from *B. abortus*-stimulated platelets (*p* < 0.005) (Figure 7A). Moreover, our results showed that cytokine and chemokine secretion of activated HBMECs is also regulated by the Erk1/2 pathway, since the inhibition with PD98059 also partially diminished the secretion of IL-6, IL-8, and CCL-2, compared to non-treated cells (Figure 7B–D). These results indicate that the Erk1/2 pathway is involved in HBMEC activation by supernatants from *B. abortus*-activated platelets.

The involvement of the Erk1/2 pathway in ICAM-1 upregulation is particularly interesting since ICAM-1 is one of the immunoglobulin-like cell adhesion molecules implicated in the transendothelial migration of immune cells [22]. Thus, we investigated the involvement of the Erk1/2 pathway on the increasing transendothelial migration throughout HBMECs activated by supernatants from *B. abortus*-stimulated platelets. For this, HBMEC monolayers on Transwells were pre-treated with PD98059 2 h before and throughout treatment with *B. abortus*-stimulated platelet supernatants. 24 h later, neutrophils were seeded in the upper chamber for 3 h and the number of migrated cells to the lower chamber was quantified. Neutrophil migration was totally inhibited in HBMECs pre-treated with PD98059, demonstrating the implication of the Erk1/2 pathway on the increased transmigration of immune cells (Figure 7E).

## 3. Discussion

Physiological and pathological immune responses are a continuum in which platelets are recognized as innate immune effector cells. Their activation stimulates interactions with endothelial cells and myeloid leukocytes in many pathologic inflammatory syndromes, as well as consequences in acute inflammation [28,29,30,31]. Platelets also have signaling functions in endothelial cells. These functions also contribute to critical inflammatory and immune responses [32,33].

Brain microvascular endothelium activation and BBB dysfunction is a significant contributor to the pathogenesis of a variety of brain pathologies [2], many of them of microbial origin [9,18,34]. We have previously described the ability of *B. abortus* to induce inflammation in the cerebral parenchyma, which leads to the activation of the endothelial cells that form the BBB [9]. In this study, we elucidated the role of platelets in brain microvascular endothelial cell activation mediated by *B. abortus*. Platelets enhance HBMEC activation in the context of *B. abortus* infection. These results correlate with the reported ability of other bacterial species to activate platelets and harm endothelial cells [35,36].

Interestingly, HBMEC activation does not require direct contact between platelets and brain endothelial cells, since supernatants of *B. abortus*-stimulated platelets recapitulated the HBMEC activation observed in the presence of platelets. Furthermore, HBMEC activation by secreted factors from *B. abortus*-stimulated platelets is sufficient to induce transmigration of both monocytes and neutrophils. Moreover, *B. abortus*-stimulated platelets also activate the HMEC-1 cell line and primary culture of HUVEC, underscoring the ability of *B. abortus*-stimulated platelets to activate any endothelium.

A long time ago, it was demonstrated that activated platelets increase CCL-2 secretion and ICAM-1 expression on HUVECs [37]. This indicates that activated platelets are able to change the chemotactic and adhesive properties of endothelial cells, increasing the ability to attract monocytes and neutrophils. Under physiological conditions, endothelial cells of the vasculature of non-inflamed tissues have as main functions the maintenance of blood fluidity and the control of vascular permeability [33]. Under these conditions, resting endothelial cells do not interact with circulating leukocytes since the proteins necessary for this interaction are mainly retained inside the cell [38]. Under acute inflammatory conditions, such as those induced by *B. abortus* infection, the vascular endothelium is rapidly activated, mobilizing these adhesion molecules to the extracellular membrane [33]. In accordance with this, we demonstrated that, although the infection with *B. abortus* induces a mild activation of HBMEC, HMEC-1, and HUVEC, the presence of platelets during the infection enhances its activation state upregulating the expression of ICAM-1 and CD40, thus stressing the amplifying role of platelets on endothelial inflammation [39]. In line with these results, other authors have shown that the presence of activated platelets significantly induces the expression of E-Selectin (CD62E), CD106 (VCAM-1), and ICAM-1 on the surface of HUVEC cells, even in the absence of others inflammatory agents [40]. In addition to the increase in adhesion molecules, we have demonstrated that the activation of HBMECs by supernatants from *B. abortus*-stimulated platelets increase IL-6, IL-8, and CCL-2 secretion. These results are in agreement with those previously published describing that HUVEC secrete IL-8 and CCL-2 after co-incubation with activated platelets [40]. In turn, in vivo experiments have shown that platelets are one of the first cellular components arrested in the inflamed endothelium, promoting their activation and allowing the subsequent arrest of leukocytes [41].

Platelet activation was also induced by exposure to heat-killed *B. abortus*, which indicated that it was not dependent on bacterial viability and suggests that it was elicited by a structural bacterial component. Our laboratory has been investigating for years the role of lipoproteins in inflammation generated by *Brucella*. We have described that *Brucella* LPS does not produce cellular activation, however, *Brucella* lipoproteins produce activation of several cell types [6,8,25,26]. Thus, we hypothesized that *B. abortus* lipoproteins might be the structural components involved in the observed phenomenon. L-Omp19, a prototypical *B. abortus* lipoprotein, recapitulated platelet stimulation and concomitant HBMEC activation. Acylation of Omp19 was required for its biological activity since U-Omp19 had no effect on platelet stimulation. The genome of *B. abortus* possesses no less than 80 genes encoding putative lipoproteins [42], and many of them are expressed in the outer membrane of the bacterium [43]. In this context, we posit that any surface-exposed *Brucella* lipoprotein may be significant beyond in vitro assays and not one lipoprotein but rather a combination of them may contribute to the platelet activation elicited by *B. abortus*.

**Figure 8:**
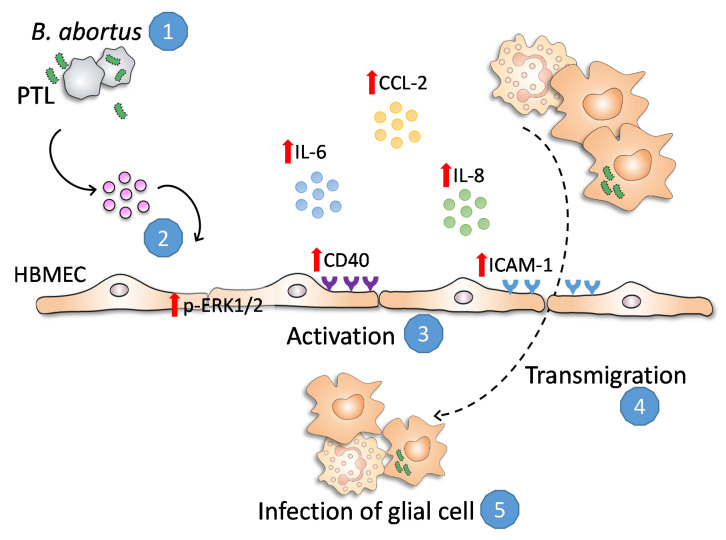
*B. abortus*-stimulated platelets (1) secret factors (2) that induce HBMEC activation, leading to ICAM-1 and CD40 upregulation, increasing the secretion of IL-6, IL-8, and CCL-2 (3), and promoting neutrophils and monocytes to traverse a polarized HMBEC monolayers (4). Platelet-induced activation would escalate the entering of infected cells from the peripheral circulation and the subsequent infection of glial cells (5), worsening the inflammatory signs of neurobrucellosis.

Our recent work revealed a physiological mechanism employed by *B. abortus* to traverse the BBB. *Brucella* is incapable of traversing the BBB by itself, despite the ability to invade and replicate in endothelial cells of the brain microvasculature. Instead, it could cross a BBB model in vitro as a consequence of naturally migrating monocytes carrying viable bacteria, which serve as source of de novo infection to astrocytes and microglia [5]. Interestingly, we have also demonstrated that activated *B. abortus*-infected glial cells were able to increase the transmigration of monocytes through the secretion of inflammatory mediators [9]. These mediators would escalate the entering of infected cells from the peripheral circulation, increasing the infection and the subsequent BBB dysfunction through a pathological vicious circle. The capacity of secreted factors from *B. abortus*-stimulated platelets to increase neutrophil and monocyte transmigration through microvascular endothelial cells demonstrated in this paper would worsen this situation (Figure 8).

The mitogen-activated protein kinase (MAPK) pathway has been associated to several biological processes such as cell activation and proliferation, cell differentiation, and apoptosis [44]. In particular, the Erk1/2 pathway is involved in HBMEC activation [27] and endothelial permeability [45]. Experiments of pharmacological inhibition determined that Erk1/2 was involved in HBMEC activation induced by supernatants from *B. abortus*-activated platelets. In particular, it was involved in ICAM-1 upregulation and enhanced the transmigration of neutrophils. Since MAPK inhibitors, such as pyridinyl imidazole drugs, have been identified as putative drugs for anti-inflammatory therapies in the CNS [46], the data presented in this paper suggest that inhibiting such molecules (Erk1/2) may represent a pharmaceutical strategy to restrict BBB deterioration, thereby potentially reducing the morbidity associated with neurobrucellosis.

In summary, the results presented here describe a mechanism whereby *B. abortus*-stimulated platelets can induce HBMEC and other endothelial cell activation, promoting neutrophils and monocytes to traverse the BBB. Moreover, this could contribute to increase the infection of glial cells, generating and/or deteriorating neurobrucellosis and the inflammatory response motivated by glial activation (Figure 8).

## 4. Materials and Methods

### 4.1. Ethics Statement

Human platelets, monocytes, and neutrophils were isolated from the blood of healthy adult donors in agreement with the guidelines of the Ethical Committee of the Instituto de Medicina Experimental (protocol number: 20160518-M). All adult blood donors provided their informed consent prior to the study.

### 4.2. Bacteria and Lipoproteins

*B. abortus* S2308 was cultured in tryptic soy broth supplemented with yeast extract (Merck, Buenos Aires, Argentina). The number of bacteria on stationary-phase cultures was determined by comparing the optical density at 600 nm with a standard curve. All live *Brucella* manipulations were performed in biosafety level 3 facilities located at the Instituto de Investigaciones Biomédicas en Retrovirus y SIDA (INBIRS, Buenos Aires, Argentina). To obtain heat-killed *B. abortus* (HKBA), bacteria were washed five times for 10 min each in sterile phosphate-buffered saline (PBS), heat-killed at 70 °C for 20 min, aliquoted, and stored at −70 °C until used. The total absence of *B. abortus* viability after heat killing was verified by the absence of bacterial growth on tryptic soy agar.

*B. abortus* lipidated-outer membrane protein 19 (L-Omp19) and unlipidated-Omp19 (U-Omp19) were obtained as described [31]. Both recombinant proteins contained less than 0.25 endotoxin U/μg of protein as assessed by Limulus Amebocyte Lysates (Associates of Cape Cod Inc., Falmouth, MA, USA).

### 4.3. Cell Lines

HBMECs were isolated from a brain biopsy of an adult female with epilepsy as previously described [47]. These cells were positive for factor VIII–Rag, carbonic anhydrase IV, and Ulex europaeus agglutinin I. They took up fluorescently labeled low-density lipoprotein and expressed g-glutamyl transpeptidase, thus demonstrating their brain endothelial cell properties [47]. HBMECs were subsequently immortalized by transfection with SV40 large T Ag and maintained their morphological and functional characteristics for at least 30 passages [48]. The cells are polarized and exhibit a transendothelial electric resistance (TEER) of at least 100 ohms/cm^2^ [49]. Cells (passage < 30) were cultured in tissue culture flasks in Roswell Park Memorial Institute (RPMI) medium 1640 (Life Technologies, Grand Island, NE, USA) supplemented 10% with heat-inactivated fetal bovine serum (FBS) (Life Technologies), 10% NuSerum IV (Becton Dickinson, Bedford, OH, USA), 1% modified Eagle’s medium nonessential amino acids (Life Technologies), sodium pyruvate (1 mM), L-glutamine (2 mM), 1% MEM vitamin solution (Life Technologies), penicillin (100 U/mL) and streptomycin (100 µg/mL). Human microvascular endothelial cells (HMEC-1) were obtained from ATCC^®^ (CRL-3243™, Manassas, VA, USA). Cells were grown in Dulbecco′s Modified Eagle’s (DMEM) medium (Life Technologies) containing 10% FBS (Natocor, Córdoba, Argentina), 10 µg/mL hydrocortisone, 1 ng/mL epidermal growth factor (BD Pharmingen, San Diego, CA, USA), L-glutamine (2 mM), penicillin (100 U/mL), and streptomycin (100 µg/mL). All cell cultures were incubated at 37 °C in a humidified atmosphere of 5% CO_2_. Human umbilical vascular endothelial cells (HUVECs) were obtained as described previously [10,50]. Briefly, umbilical vascular tissue was treated with collagenase for digestion. Cells were seeded until confluence on 1% gelatin-coated 25 cm^2^ tissue culture flasks and identified by their cobblestone morphology and von Willebrand factor (VWF) antibody (Immunotech, Ocala, FL, USA) binding. Cells were grown in RPMI 1640 medium (Gibco) supplemented with 10% FBS (Life Technologies), heparin (100 µg/mL), endothelial cell growth factor (50 µg/mL), sodium pyruvate (2 mM), L-glutamine (2 mM), penicillin (100 U/mL), and streptomycin (100 µg/mL) at 37 °C in a humidified 5% CO_2_ incubator. HUVECs used for experiments were kept between the first and third culture passage.

### 4.4. Platelet Purification and Stimulation

Platelets were obtained from whole blood from healthy adult human donors as described previously [14]. Briefly, blood samples were collected into tubes containing sodium citrate (Merck) and centrifuged. The platelet-rich plasma was collected and centrifuged in presence of 75 nM prostaglandin I2 (Cayman Chemical, Ann Arbor, MI, USA). Platelets were then washed with RPMI 1640 medium. Finally, platelets were resuspended in RPMI 1640 medium. Platelets were incubated with *B. abortus* (1 × 10^7^/mL) (PLT:B.a. ratio of 1:1) for 24 h in RPMI 1640 medium with 10% FBS (Life Technologies) and L-glutamine (2 mM). In addition, platelets were incubated with HKBA (1 × 10^8^ bacteria/mL), L-Omp19, U-Omp19 (both 500 ng/mL), or thrombin (0.1 U/mL) (Sigma Aldrich, St. Louis, MO, USA). Then, supernatants were collected, sterilized by filtration, ultracentrifuged when mentioned (at 100,000× *g* for 5 h at 4 °C), and stored at −70 °C until they were used.

### 4.5. Endothelial Cell Treatment

HBMEC, HMEC-1, and HUVEC were cultured in 48 wells plate (5 × 10^4^/0.2 mL). To co-culture infection, platelets were added (cell:platelets ratio, 1:100) and endothelial cells–platelets cultures were infected by *B. abortus* (multiplicity of infection of 100). In all cases, the infection was performed for 2 h in medium containing no antibiotics. Then, cells were maintained for 24 h in the presence of antibiotics (100 µg/mL gentamicin and 50 µg/mL streptomycin) to kill the remaining extracellular bacteria. For experiments with platelet-conditioned media, HBMEC, HUVEC, and HMEC-1 cells were treated with 0.2 mL of diluted supernatants from *B. abortus*-stimulated platelets for 24 h. Culture supernatants from *Brucella*-infected astrocytes and recombinant human IL-1β were used as control. In all cases, cells were harvested to determine cell surface molecule expression by flow cytometry. Supernatants from stimulated endothelial cells were collected and stored at −70 °C until they were used.

### 4.6. Erk1/2 Signaling Pathway

HBMECs were treated with Erk1/2 MAPK pharmacological inhibitor PD98059 (50 μM) (Calbiochem, San Diego, CA, USA) or vehicle (dimethyl sulfoxide) 2 h before the stimulation with supernatants and the inhibitor were kept throughout the experiment, based on previous report [7].

### 4.7. Measurement of Cytokine and Chemokine Concentrations

Human IL-6, IL-8, and CCL-2 concentrations were quantified in supernatants harvested from HBMECs and HMEC-1 treated with supernatants from *B. abortus*-stimulated platelets by Sandwich ELISA using paired cytokine-specific mAbs according to the manufacturer’s instructions (BD Pharmingen).

### 4.8. Determination of Cell Surface Molecules by Flow Cytometry

ICAM-1 and CD40 surface expression was determined by flow cytometry. For this, treated HBMECs or HMEC-1 were washed and stained with a PE-labeled antibody (Ab) against human ICAM-1 (CD54) (clone HA58, BD Pharmingen), PE-labeled Ab against human CD40 (clone 5C3; BioLegend, San Diego, CA, USA) or the PE-labeled isotype-matched control Ab (BD Pharmingen). Labeled cells were analyzed on a FACSCalibur flow cytometer (BD Biosciences, San Diego, CA, USA), and data were processed using FlowJo software.

### 4.9. Neutrophil and Monocytes Transendothelial Migration Assay

Peripheral blood mononuclear cells (PBMCs) and neutrophils were separated by Ficoll-Hypaque (GE Healthcare, Uppsala, Sweden) gradient centrifugation. Human neutrophils were isolated by sedimentation of erythrocytes in 6% dextran and hypotonic lysis as previously described [26]. Monocytes were then purified from PBMCs by Percoll (GE Healthcare) gradient. Both types of cells were resuspended in RPMI 1640 supplemented with 10% FBS. Cell purity was 90% as determined by flow cytometry for both populations. Viability of cells was more than 95% in all the experiments as measured by trypan blue exclusion test.

HBMEC monolayers were established from 20,000 cells per insert on 3-μm pore size membrane Transwell plates of 6.5-mm diameter insert (Corning-Costar, Acton, MA, USA) previously treated with rat tail collagen (50 mg/mL in 1% acetic acid) (BD Biosciences) and neutralized in a saturated atmosphere of ammonium hydroxide. After 5 days, when cellular confluence was reached TEER and passive diffusion of horseradish peroxidase was measured as an indication of monolayer integrity [5]. Then, monolayers were incubated for 24 h with supernatants from *B. abortus*-stimulated platelets. Supernatants from platelets alone as well as non-treated HBMECs were used as negative control. Culture supernatants from *Brucella*-infected astrocytes and recombinant human IL-1β were used as positive control. After that, monolayers were washed and neutrophils or monocytes (1 × 10^5^ cells) were added to the upper chamber in fresh medium. Plates were incubated for 3 h at 37 °C in 5% CO_2_ and transmigrated cells to the lower chamber were counted on a hemocytometer.

### 4.10. Statistical Analysis

Results were analyzed with one-way ANOVA followed by Tukey post-test using the GraphPad Prism 5.0 software.
